# *In vivo* biocompatibility, clearance, and biodistribution of albumin vehicles for pulmonary drug delivery

**DOI:** 10.1016/j.jconrel.2015.05.269

**Published:** 2015-07-28

**Authors:** A. Woods, A. Patel, D. Spina, Y. Riffo-Vasquez, A. Babin-Morgan, R.T.M. de Rosales, K. Sunassee, S. Clark, H. Collins, K. Bruce, L.A. Dailey, B. Forbes

**Affiliations:** a Drug Delivery Research Group, Institute of Pharmaceutical Science, King's College London, 150 Stamford Street, London, SE1 9NH, United Kingdom; b Division of Imaging Sciences and Biomedical Engineering, King's College London, 4th Floor Lambeth Wing, St Thomas' Hospital, London SE1 7EH, United Kingdom; c Sackler Institute of Pulmonary Pharmacology, Institute of Pharmaceutical Science, King's College London, 150 Stamford Street, London SE1 9NH, United Kingdom; d Division of Immunology, Infection & Inflammatory Diseases, Guy's Campus, King's College London, 15-16 Newcomen Street, London SE1 1UL, United Kingdom

**Keywords:** Albumin nanoparticles, SPECT/CT, Alveolar macrophages, Nanomedicine, Biodistribution, Pulmonary drug delivery

## Abstract

The development of clinically acceptable albumin-based nanoparticle formulations for use in pulmonary drug delivery has been hindered by concerns about the toxicity of nanomaterials in the lungs combined with a lack of information on albumin nanoparticle clearance kinetics and biodistribution. In this study, the *in vivo* biocompatibility of albumin nanoparticles was investigated following a single administration of 2, 20, and 390 μg/mouse, showing no inflammatory response (TNF-α and IL-6, cellular infiltration and protein concentration) compared to vehicle controls at the two lower doses, but elevated mononucleocytes and a mild inflammatory effect at the highest dose tested. The biodistribution and clearance of ^111^In labelled albumin solution and nanoparticles over 48 h following a single pulmonary administration to mice was investigated by single photon emission computed tomography and X-ray computed tomography imaging and terminal biodistribution studies. ^111^In labelled albumin nanoparticles were cleared more slowly from the mouse lung than ^111^In albumin solution (64.1 ± 8.5% *vs* 40.6 ± 3.3% at t = 48 h, respectively), with significantly higher (P < 0.001) levels of albumin nanoparticle-associated radioactivity located within the lung tissue (23.3 ± 4.7%) compared to the lung fluid (16.1 ± 4.4%). Low amounts of ^111^In activity were detected in the liver, kidneys, and intestine at time points > 24 h indicating that small amounts of activity were cleared from the lungs both by translocation across the lung mucosal barrier, as well as mucociliary clearance. This study provides important information on the fate of albumin vehicles in the lungs, which may be used to direct future formulation design of inhaled nanomedicines.

## Graphical abstract

**Figure:**
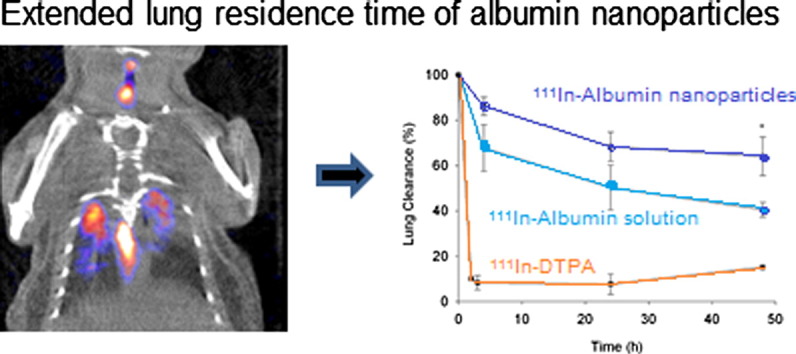

## 1 Introduction

The potential for nanoparticles to provide controlled drug delivery to the lungs is well recognised [1], [2]. The benefits of using nanoparticles as oppose to micron-scale carriers in the lungs include the ability to reduce unwanted macrophage uptake [3], modify dissolution rates particularly of poorly soluble drugs [4], and to improve deposition and retention profiles in the lungs [5], [6]. However, there has been little translation of pulmonary nanomedicine from the research bench to the clinic, not least because of safety concerns associated with inhalation of nanoparticulate matter [7], [8].

Chronic exposure to certain types of nanoparticles has been associated with inflammation, fibrosis and the release of pro-thrombotic mediators which can lead to induced vascular thrombosis and cardiac arrest [9]. In addition, inhaled nanoparticles have been observed to pass through the lung epithelium and enter the systemic circulation prompting fears concerning accumulation in secondary organs [10]. The type and extent of toxicity elicited by a particulate nanocarrier has been linked to a variety of factors including particle size, composite material, surface charge and reactivity, hydrophobicity, and aspect ratio [11], [12], [13], [14], [15]. In addition to acute inflammatory response, insoluble nanoparticles are associated with long retention times in the lungs making accumulation associated toxicity upon repeated dosing a concern [16]. Any candidate as a medical nanoparticle formulation for pulmonary administration must therefore be inert, biodegradable, and biocompatible both at the nanoscale and in terms of breakdown products [17], [18].

Albumin, both in its native form and when fabricated into nanoparticles, is used with great success and versatility as a drug carrier in injected formulations [19], [20]. An albumin nanoparticle based-formulation, Abraxane®, is used clinically to deliver the highly potent anti-cancer drug, paclitaxel, without the need for stabilizing agents [21]. With regard to its suitability for pulmonary drug delivery, albumin is an abundant component of lung lining fluid [22], with a concentration ~ 10% of that found in serum [23]. As albumin is naturally present within the lungs, endogenous transport and metabolic processes exist which could be utilised for drug release [24], [25] and alleviate concerns about long term accumulation associated toxicity. Self-assembling human serum albumin nanoparticles have been recently developed as a novel vehicle for a combination formulation consisting of doxorubicin and tumour necrosis factor related apoptosis-inducing ligand (TRAIL). This formulation was shown to significantly reduce lung tumour size following pulmonary administration in a mouse model of lung cancer [26]. In addition, the ^99m^Tc-labelled aggregated albumin complex ^99m^Tc-Nanocoll® has been used extensively for diagnostic imaging in nuclear medicine and to determine pulmonary deposition patterns in human subjects following aerosol inhalation [27], [28].

Despite the interest in developing albumin nanoparticles for pulmonary drug delivery, information on the safety, clearance kinetics, and biodistribution of the albumin vehicle is currently lacking. It is recognised that the highest achievable therapeutic dose of an inhaled nano-formulation may be limited by the highest tolerated dose of the nanomaterial rather than that of the therapeutic compound. Secondly, estimation of nano-formulation pharmacokinetics and administration frequency must include an understanding of the vehicle clearance kinetics as well as that of the therapeutic agent [29]. Therefore, the aim of this study was to evaluate the dose-dependent biocompatibility of two albumin vehicles (solution and nanoparticles) following a single pulmonary administration to mice *in vivo*. Clearance of ^111^indium-diethylene triamine pentaacetic acid (^111^In-DTPA) radiolabelled albumin vehicles from the lungs of mice was also measured longitudinally using combined single photon emission computed tomography and X-ray computed tomography (SPECT/CT) imaging over 48 h. Local distribution of albumin and albumin nanoparticles in the lung, as well as accumulation in secondary organs was also measured using terminal end-point organ harvest biodistribution studies. The provision of information on albumin vehicle biocompatibility, clearance kinetics and biodistribution will ultimately enable formulation scientists to decide whether the use of an albumin vehicle is an appropriate tool to achieve a desired pharmacokinetic profile for an intended therapy prior to engaging in lengthy formulation optimisation studies. Secondly, knowledge of the maximum tolerated dose of the albumin vehicle will enable estimations of the theoretical drug loading requirements, providing the basis for a go/no-go decision-making tool in nano-formulation design.

## 2 Methods

### 2.1 Materials and reagents

Bovine serum albumins (laboratory grade albumin, albumin tissue culture grade and low endotoxin, essentially IgG free albumin) were purchased from Sigma (Dorset, UK). S-2-(4-Isothiocyanatobenzyl)-diethylenetriamine pentaacetic acid (*p*-SCN-Bn-DTPA) was purchased from Macrocyclics (Dallas, USA). ^111^InCl_3_ was purchased from Mallinckrodt Medical Inc. (Petten, Netherlands). Phosphate buffered saline (PBS) tablets were purchased from Oxoid (Basingstoke, UK) and prepared using ultrapure water according to the manufacturer's instructions. Foetal bovine serum was purchased from Life Technologies (USA). Diethylenetriamine pentaacetic acid (DTPA) and ammonium acetate were purchased from Fluka (UK). Sodium hydroxide (NaOH) and Tris(hydroxymethyl)aminomethane (Tris) were purchased from Fisher Scientific (Loughborough, UK). Glutaraldehyde (50% vol/vol in water) was purchased from Aldrich (UK). Ethanol was purchased from VWR (Radnor, USA).

### 2.2 Animals

All experiments were conducted in accordance with the United Kingdom Animal Scientific Procedures Act, 1986 and were approved by the ethics committee of Kings College, London. All *in vivo* experiments were performed with Male BALB/C mice (6–8 weeks of age; approx 20–26 g) which were purchased from Harlan, UK. Mice were housed in rooms maintained at a constant temperature (21 ± 2 °C) and humidity (55 ± 15%) with a 12 hour light–dark cycle. Animals had food (SDS, UK) and water available *ad libitum* and were allowed 1 week acclimatisation period before use.

### 2.3 Nanoparticle preparation and characterization

Nanoparticles were prepared using a desolvation method as widely reported in the literature [30], [31]. In brief, bovine serum albumin (100 mg) was dissolved in 0.01 M Tris HCl buffer (1 mL) and the pH adjusted to 9 with 1 M NaOH. Ethanol was added drop-wise to the stirred protein (4.0 mL, 1 mL/min). Nanoparticles were cross-linked with 10% vol/vol glutaraldehyde/water (47.2 μL) and stirred overnight at room temperature, and purified into sterile PBS by four cycles of spin filtration (30 kDa molecular weight cut-off (MWCO) centrifugal filter units, Millipore, 2400 ×*g*, 10 min per cycle). Nanoparticle size and polydispersity were measured using a Zetasizer Nano Series ZS (Malvern Instruments, Malvern, UK). Particles were diluted 1:10 in PBS and measurements taken at 37 °C, scattering angle 173°, using viscosity value of 0.6885 cP for the dispersant. Zeta potential was measured in PBS at 25 °C at a concentration of 20 μg/mL. Particle concentration was determined gravimetrically.

### 2.4 *In vitro* macrophage activation assays

Albumin nanoparticles were prepared using three grades of bovine serum albumin: laboratory grade (> 96% purity); tissue culture grade; low endotoxin grade (all Sigma, UK). J774.A1 cells, a macrophage cell line derived from BALB/C mice, were seeded in 24-well plates at a density of 1 × 10^6^ cells per well and incubated overnight in cell culture medium (DMEM, 4.5 g/L glucose, 1% sodium pyruvate, 1% penicillin/streptomycin, 1% HEPES buffer and 1% l-glutamine) containing 10% (v/v) foetal bovine serum (FBS) in a humidified 5% CO_2_: 95% air incubator at 37 °C. Following incubation, cell culture medium was replaced with 200 μL of fresh medium containing 4350 μg/mL of albumin nanoparticles prepared from each BSA grade. Positive control cells were primed for 18 h with interferon-gamma (IFN-γ) prior to incubation with 1 μg/mL lipopolysaccharide (from *Escherichia coli* 0111:B4; Sigma Aldrich, UK). Untreated cells were used as a negative control. Cells were incubated with nanoparticles for 4 h, following which 100 μL supernatant was removed and analysed to quantify levels of interleukin-6 (IL-6), tumour necrosis factor alpha (TNF-α) and nitric oxide. TNF-α and IL-6 release were measured using enzyme-linked immunosorbent assay (eBioscience, conducted according to manufacturer's instructions). Nitric oxide (NO) production was measured using the Griess reaction [32].

### 2.5 *In vivo* biocompatibility

Low endotoxin grade albumin nanoparticles were suspended in sterile phosphate buffered saline (PBS) and administered at three doses for biocompatibility studies, 2, 20, and 390 μg per mouse (~ 0.1, 1, 16 mg/kg). The doses fall within the range typically used for inhalation studies of this nature [15], [33], [34]. Sterile PBS was used as a vehicle control. Albumin nanoparticles were administered *via* oropharyngeal aspiration (o.a.; method adapted from Lakatos et al., 2006 [35]). Briefly, anaesthetized mice (3–5% isoflurane, O_2_ flow rate of 1.0 mL/min) were administered a droplet of 25 μL of albumin nanoparticle suspension in sterile saline (2, 20 or 390 μg/mouse) applied to the posterior base of the pharynx, where it was readily aspirated. Mice were euthanized humanely after 24 h with an intraperitoneal (i.p.) injection of urethane (500 mg/mL in physiological saline, 0.3 mL/mouse). The lungs were lavaged with 3 × 0.5 mL sterile physiological saline. The total number of cells in the lavage fluid was counted using a Neubauer haemocytometer (Fisher Scientific, Loughborough, UK). Diffquick® (DADE Behring, Marburg, Germany) staining was used to perform differential cell counts in 100 cells per mouse. Eosinophils were not observed in the BAL fluid, so only macrophages and neutrophils have been reported. Total protein content in BAL was quantified using a Quick Start™ Bradford Protein Assay kit (Bio-Rad, Hemel Hempstead, UK) according to the manufacturer's instructions. Levels of BAL IL-6 and TNF-α were quantified using a mouse IL-6 or TNF-α ELISA kit (Thermo Scientific, Rockford, USA) according to the manufacturer's instructions. Tissue histology was performed as described previously by Egger et al. [36]. In brief, three transverse sections (~ 2 mm thick) were cut through the left lung (superior, median, and caudal regions), which had been fixed in 10% formalin for at least one week prior to tissue processing. Lung sections were dehydrated through ascending grades of ethanol and embedded in paraffin wax. Histological slices (4 μm) were obtained from each lung section and stained with haematoxylin and eosin (H&E) to assess general morphology.

### 2.6 Preparation and serum stability of ^111^In labelled albumin nanoparticles

^111^In labelled albumin was prepared by first synthesizing 2-(4-isothiocyanatobenzyl)-diethylenetriaminepentaacetic acid-conjugated bovine serum albumin (*p*-SCN-Bn-DTPA-albumin), followed by labelling with ^111^In. Labelled albumin nanoparticles were prepared from ^111^In labelled protein using a modified, small scale desolvation method (mini-prep). Briefly, bovine serum albumin (33 mg) was dissolved in ice cold 0.1 M sodium carbonate buffer (pH 9.2, 10 mL). *p*-SCN-Bn-DTPA was dissolved in dry DMSO (3 mg/mL, 50 μM), added drop-wise to the stirred protein solution on ice, and left to stir overnight at 4 °C. Excess *p*-SCN-Bn-DTPA was removed by four cycles of spin filtration (10 k Da MWCO, 2400 ×*g*, 10 min and 4 °C), and buffer was replaced by chilled ultrapure water (MilliQ, 18.2 mΩ). Prior to reaction with ^111^In, 10% v/v 1 M ammonium acetate buffer was added to *p*-SCN-Bn-DTPA-albumin solution to adjust the pH to 6.6 for radiolabelling. ^111^InCl_3_ (1 part) was diluted with 2 parts ammonium acetate buffer (0.5 M, pH 6.6), then incubated with an equal volume of *p*-SCN-Bn-DTPA-albumin for 30 min at 37 °C [37]. Any unbound activity was removed by at least 5 cycles of spin filtration (30 kDa, 5000 ×*g*, 3 min). Radiolabelling efficiency was calculated from activity measured in washings, bound to the filter and in final recovered product. ^111^In albumin for administration to mice as a solution was prepared in sterile PBS.

Nanoparticles were prepared by mixing 100 μL ^111^In-labelled *p*-SCN-Bn-DTPA-albumin (^111^In albumin) with Tris HCl (0.1 M) and NaOH (1 M), followed by drop-wise addition of a 4-fold volume of ethanol and rotating (500 rpm). 10% (w/v) glutaraldehyde in water was added followed by overnight incubation under rotation (500 rpm) and purification by spin filtration (100 kDa, 5000 × g, 3 min). Radiolabelling efficiency was calculated from activity measured in the total waste, filters and in the recoverable product. Radiolabel stability in biological media was evaluated by incubating aliquots of ^111^In albumin nanoparticles and solution with foetal bovine serum (FBS) at 37 °C for 48 h (n = 5). At t = 24 and 48 h, 250 μL samples were removed, centrifuged 3 min at 10000 ×*g* using 30 kDa MWCO spin filters (Millipore), the supernatant and filtrate collected and activity in each measured using a gamma counter (1282 Compugamma Laboratory Gamma Counter, LKB Wallac) to determine the proportion of activity leached from the sample.

### 2.7 SPECT/CT 3D imaging

Healthy male BALB/C mice were anaesthetized with isoflurane (level 2–2.5, O_2_ flow rate 0.8 mL/min), and administered 25 μL of either ^111^In albumin nanoparticles or solution in sterile PBS (300–380 μg per mouse, activity matched 1–3 MBq per mouse, n = 3 per treatment group) *via* o.a. administration. Mice were placed in a prone position on the NanoSPECT/CT PLUS preclinical animal scanner (Mediso, Hungary) and imaged immediately post-administration. CT images were obtained with 55 kVP X-ray source, 500 ms exposure time in 240 projections, with a pitch of 1 over approximately 8 min. Immediately following CT scan, SPECT images were acquired using an exposure time of 1200 s, obtained over 60 projections (60 s per projection) and equipped with a 4-head scanner each with nine 1 mm pinhole apertures in helical scan mode with a total acquisition time of 35 min. CT images were reconstructed in a 352 × 352 matrix using Bioscan InVivoScope (Bioscan, USA) software, whereas SPECT images were reconstructed in a 256 × 256 matrix using HiSPECT (ScivisGmbH, Bioscan). Images were fused and analysed using InVivoScope (Version 1.44, Bioscan). Further SPECT/CT scans were acquired at t = 4 h, 24 h, and 48 h post-dosing. Quantification of activity in organs of interest was performed using the quantification tool of the InVivoScope software, selecting each organ (lungs, mouth, trachea, liver, intestines) as a region of interest (ROI). Activity in lungs during the first scan (t = 0.25 h) was taken as 100% activity for clearance kinetics calculations. Mice dosed with ^111^In-DTPA in PBS were used as controls and were treated as above with the exception of additional images collected at t = 0, 11, 23, 35, 120, and 360 min to capture the faster rate of clearance from the lung. Following the final scan, mice were culled by cervical dislocation and the trachea, throat, lungs, heart, liver, intestines, spleen, kidneys, bladder, muscle, and blood were collected for biodistribution studies.

### 2.8 BAL analysis and organ biodistribution studies

Mice were treated with ^111^In albumin nanoparticles and ^111^In albumin (180–260 μg per mouse, n = 3–4 per time point, 0.2–0.5 MBq) *via* o.a. administration. At t = 4, 24, and 48 h (n = 3–5 for each treatment group), mice were humanely culled and BAL fluid collected as described above. Activity was measured in a 200 μL aliquot of BAL and the remaining fluid was centrifuged 20 min at 1000 ×*g*. The cellular fraction was separated from the fluid phase and resuspended in PBS (1 mL). Activity in lung tissue, whole BAL (200 μL), supernatant and cellular fraction was measured by gamma scintigraphy. Following lung lavage, the trachea, throat, lungs, heart, thymus, liver, intestines, spleen, kidneys, bladder, muscle, faeces, and blood were collected and activity was measured using gamma scintigraphy. Activity in 5 μL aliquots of ^111^In albumin nanoparticles, ^111^In albumin and ^111^In DTPA preparations used for administration were measured to allow calculation of activity in organ as % of total dose.

### 2.9 Statistical analysis

BAL cell counts and differentials were analysed using GraphPad Prism 5 software (version 5.02, GraphPad Software Inc., USA) using Mann–Whitney test. Lung clearance data from SPECT/CT image analysis was performed by systematic comparison of group means using two-tailed Student's *t*-test (SigmaPlot version 12.0, Systat Software Inc., UK). BAL and lung tissue analysis was performed using Two Way ANOVA with a post-hoc Tukey's test (SigmaPlot version 12.0, Systat Software Inc, UK). P < 0.05 was considered significant.

## 3 Results

### 3.1 Nanoparticle characterization and radiolabelling

**Fig. 1:**
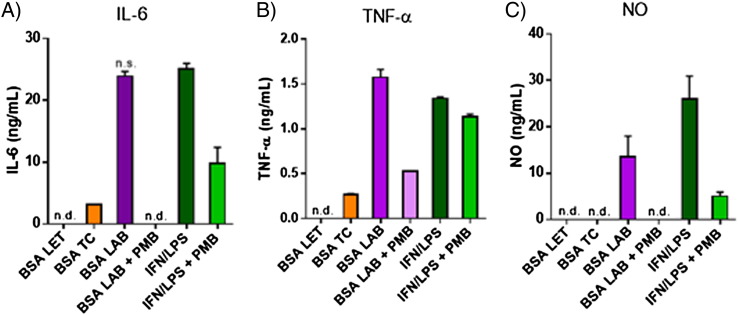
Elevated endotoxin contaminant levels present in lab-grade (BSA LAB) and tissue culture-grade (BSA TC) albumin nanoparticles stimulated macrophage activation *in vitro*, as expressed by (A) IL-6, (B) TNF-α, and (C) NO release. Low endotoxin-grade albumin nanoparticles (BSA LET) and media controls (data not shown) were non-stimulatory. Samples labelled + PMB indicate co-treatment with polymyxin B. Data represent mean ± SD (n = 3). n.d. = below the detection limit, n.s. = P > 0.05 compared to IFN/LPS positive control. All other samples differed significantly from the IFN/LPS positive control (P < 0.05).

As a quality control screen for endotoxin levels, nanoparticles were prepared from lab-grade (BSA LAB; 150 ± 32 nm), tissue culture grade (BSA TC; 171 ± 9 nm) and low endotoxin-grade (BSA LET; 108 ± 3 nm) albumin, then incubated with the murine macrophage cell line J774.A1 followed by assessment of IL-6, TNF-α and nitric oxide (NO). Co-treatment of selected samples with polymyxin B (PMB), an agent known to suppress the immunostimulatory effects of LPS [38], was used to confirm LPS contamination as the likely cause of the inflammatory response. Cells exposed to albumin nanoparticles prepared from low-endotoxin, IgG-free albumin did not release detectable levels of IL-6, TNF-α, or NO over a 4 h incubation period (Fig. 1). All subsequent experiments were therefore carried out with low endotoxin grade albumin (referred to simply as albumin in the further text).

Albumin nanoparticles prepared using the desolvation method were of a reproducible size and narrow size distribution (Table 1). ^111^In albumin nanoparticles were larger with a broader particle size distribution, compared with non-radiolabelled nanoparticles, as a result of necessary modifications to the manufacturing protocol (lower volumes and initial albumin concentrations [31]) required for successful radiolabelling. All albumin nanoparticles exhibited a moderately negative zeta potential in PBS (Table 1). The radiolabelling efficiency was 55.8 ± 21.9% with ^111^In albumin nanoparticle yields of 67.3 ± 14.1%. Serum stability studies confirmed that activity remained firmly associated with albumin (in nanoparticles or solution) over the 48 h study period.

### 3.2 *In vivo* biocompatibility

**Fig. 2:**
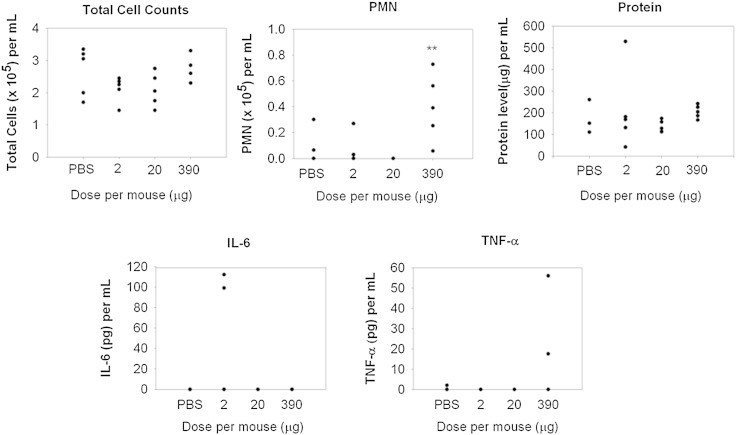
Total cell counts, polymorphonucleocytes (PMN), protein concentration and cytokine release in BAL fluid 24 h after o.a. administration of albumin nanoparticles at three dose levels. Data represent n = 5 (n = 3–5 for PBS controls). ** P < 0.01 from PBS vehicle controls.

Following o.a. administration of three different doses of albumin nanoparticles to mice, no significant change compared to vehicle control was observed at t = 24 h with regard to the total number of BAL cells, number of BAL polymorphonucleocytes (PMN) or BAL fluid protein content or IL-6 and TNF-α levels (Fig. 2). The single exception to this was an observed increase in BAL PMN at the highest dose of albumin nanoparticles.

Histopathology confirmed an absence of inflammatory response in tissue following administration of the lowest dose of albumin nanoparticles (Fig. S1), with some mild cell infiltration (macrophages and monocytes) following administration of the 20 μg dose to the mouse lung. Histopathology of lungs exposed to the highest dose of albumin nanoparticles (390 μg) revealed minor epithelial damage and low levels of neutrophilic infiltration, mirroring the observations in BAL.

### 3.3 SPECT/CT analysis of lung clearance and biodistribution

**Fig. 3:**
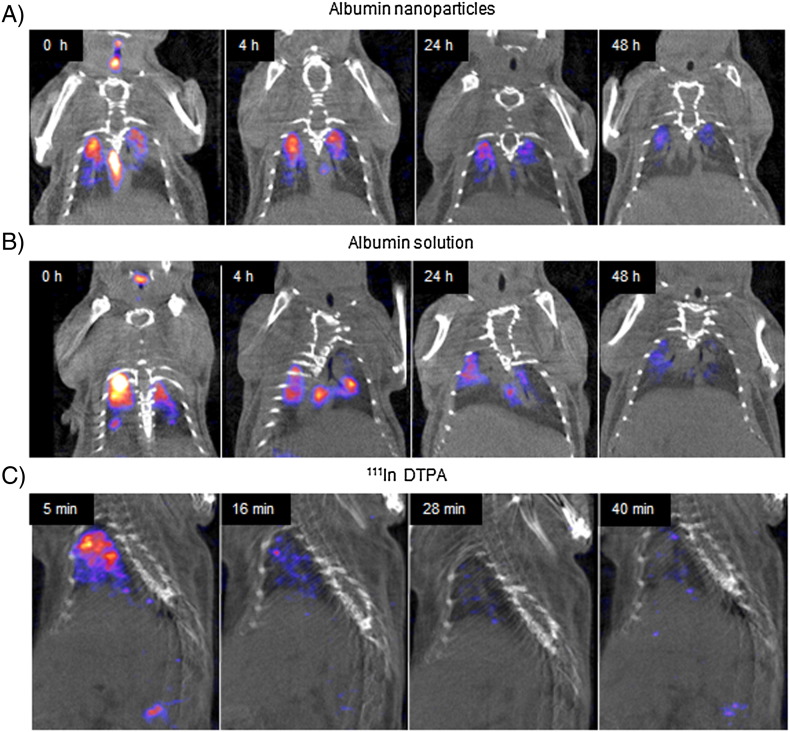
Representative SPECT/CT images showing clearance of ^111^In-albumin activity from the mouse lung over 48 h after administration of albumin (300–380 μg per animal) in the form of (A) ^111^In albumin nanoparticles and (B) ^111^In albumin solution, and (C) rapid rate of clearance of the control complex ^111^In-DTPA from the mouse lung over 40 min.

**Fig. 4:**
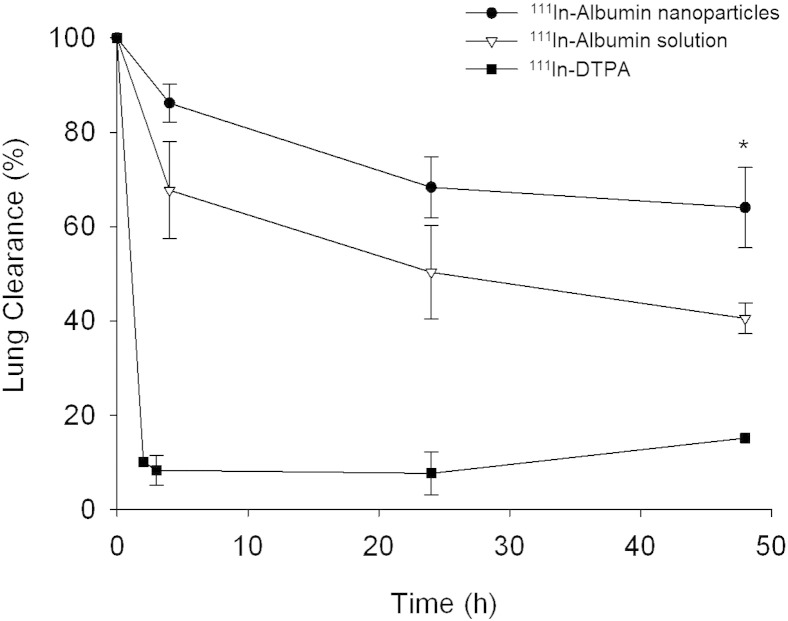
Lung clearance profiles of ^111^In-albumin nanoparticles (●),^111^In-albumin solution (▽) and ^111^In-DTPA (■) following o.a. administration (n = 3; mean ± SD). Data are decay-corrected profiles derived from SPECT/CT images and expressed as % lung clearance (relative to ^111^In activity in the lungs a t = 0). * P = 0.01 statistically significant difference from albumin solution.

SPECT/CT analysis revealed that the o.a. dosing technique delivered 12.5–38.2% of ^111^In albumin nanoparticles and 3.4–37.7% of ^111^In albumin to the lungs of mice (Fig. S2 in supplementary information). The remainder of the dose was largely found in the mouth, trachea, and intestines, indicating swallowing and subsequent clearance *via* the digestive tract over 24 h for any non-pulmonary component (Fig. S3 in supplementary information). SPECT/CT images showed that ^111^In albumin nanoparticles were retained throughout the 48 h imaging period (Fig. 3). Quantification of the SPECT/CT images revealed that ^111^In albumin nanoparticles were cleared more slowly from the lungs than ^111^In albumin over the imaging period (Fig. 4). At 48 h, a significantly (P = 0.01) higher fraction of ^111^In albumin nanoparticles (64.1 ± 8.5%) remained in the lungs compared with ^111^In albumin delivered as a solution (40.6 ± 3.3%).

Any ^111^In albumin not inhaled could be visualised passing through the GI tract in the first 4 h post-dosing, and was largely cleared from the body by the 24 h time point (Figs. 3, S3). ^111^In DTPA controls showed activity cleared extremely rapidly from the lungs with only 8.4% of the inhaled dose remaining in the lungs by 3 h. ^111^In DTPA activity was observed in the bladder and kidneys in the first hour of scanning, indicating clearance *via* the urine.

### 3.4 Organ biodistribution

**Fig. 5:**
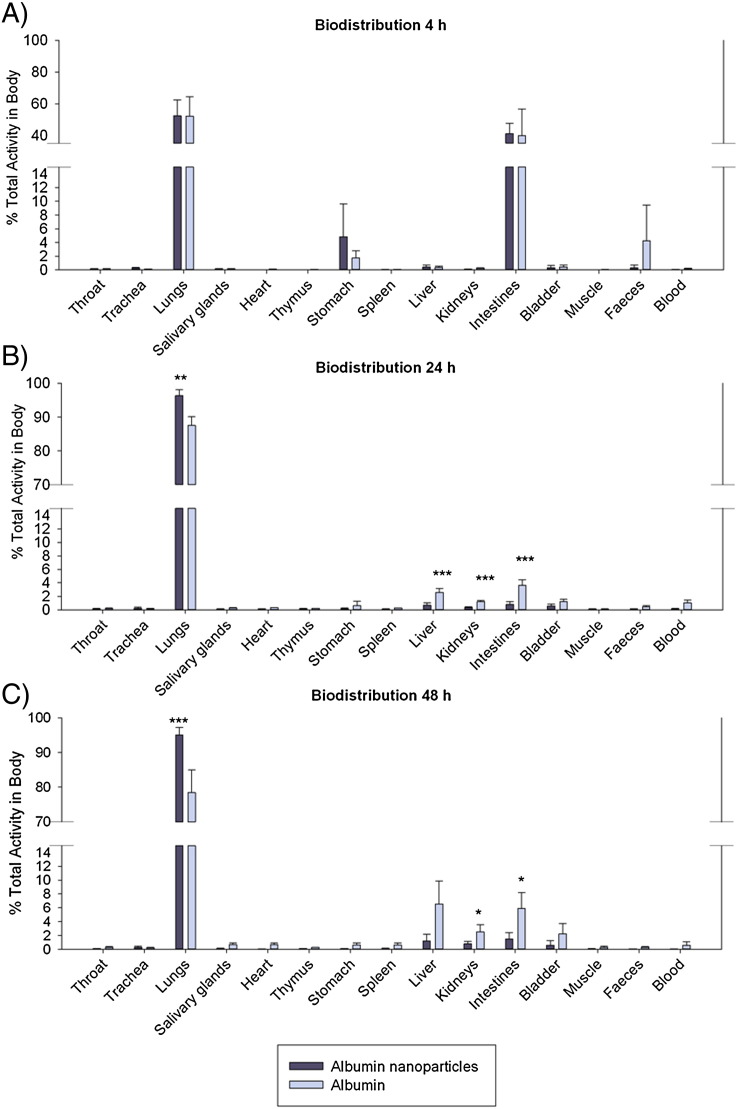
^111^In activity measured directly in major organs of mice expressed as the proportion of total activity remaining in the body and each time point: t = 4 h (n = 3), 24 h (n = 3), and 48 h (n = 6). Data represent mean ± sd. *** P < 0.001; ** P < 0.01; * P < 0.05.

Distribution of activity measured in organs at 4, 24, and 48 h post-administration of albumin nanoparticles and albumin was in good agreement with SPECT/CT imaging results at all time points. At 4 h post-administration with ^111^In albumin nanoparticles and solution, the majority of radioactivity was observed in the lungs and the GI tract (Fig. 5). Following this, the albumin solution was cleared from the lungs more rapidly, with significant albumin-associated activity found in the intestines, liver and kidneys compared to nanoparticle-associated activity, which remained primarily in the lungs. The overall agreement in results between terminal end-point organ analysis and imaging studies (*e.g.* slower clearance of albumin nanoparticles compared to solution, no major organ accumulation) provides evidence to support the case for SPECT/CT studies as a viable, animal-sparing alternative to measure particle distribution and fate [39].

Organ analysis following administration of ^111^In-DTPA was also in agreement with SPECT/CT imaging in demonstrating the rapid clearance of the label and chelator from the lungs directly into the bloodstream followed by renal clearance within 3 h (Fig. S4). This rapid absorption of ^111^In-DTPA and concentration in the urine was in stark contrast to the clearance profiles of ^111^In albumin in solution or particulate form, confirming that the label remained attached to albumin for the duration of the *in vivo* experiments.

**Fig. 6:**
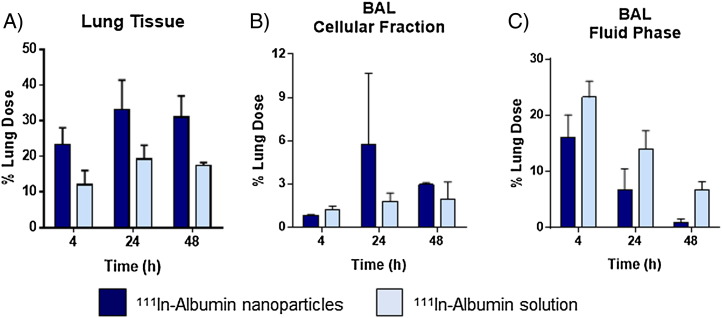
Distribution of ^111^In-albumin within murine lungs at t = 4, 24 and 48 h after o.a. administration of 180–260 μg albumin nanoparticles or albumin solution. Plots show the radioactivity expressed as percentage of the lung dose associated with (A) lung tissue, (B) cellular fraction or (C) BAL fluid phase. P < 0.001 between ^111^In-albumin nanoparticles *vs* solution in lung tissue and BAL fluid phase across all time points as determined by two-way ANOVA. No significant differences were observed between formulations in the BAL cellular fraction. Data represent mean ± SD (n = 3–4).

Analysis of lung tissue and BAL fluid revealed significant differences in the location of ^111^In albumin within the lungs over 48 h after delivery as nanoparticles compared to solution (Fig. 6). For both formulations, a substantial fraction of albumin delivered was found within the lung tissue (*i.e.* non-recoverable by BAL) within 4 h, and increased over the study period. Lung tissue accumulation was significantly higher for nanoparticulate albumin compared with albumin solution (P < 0.001) at all time points measured. Albumin-associated ^111^In activity also accumulated within the BAL cellular fraction over time, with a 2.5-fold greater cellular localization of albumin nanoparticles (12.6 ± 10.4% *vs* 4.7 ± 1.7% for solution). At all times, the activity found in the BAL fluid phase was significantly higher (P < 0.001) when delivered as ^111^In albumin solution compared to ^111^In albumin nanoparticles.

## 4 Discussion

Despite the exciting opportunities they afford, safety concerns have limited the development of nanomedicines for pulmonary use [7], [40], [41] since even materials that are relatively inert in the bulk scale may be associated with toxicity when nano-sized [42], [43]. However, there have been few studies into the safety and targeting potential of putatively well-tolerated nanoparticulate drug carriers for pulmonary delivery. In this study, albumin nanoparticles produced little or no inflammatory response *in vitro* or in mouse lungs following pulmonary delivery. Only at the highest dose administered (390 μg/lung) did any of the end-points return a positive signal, and this was limited to a mild neutrophilia (6.5-fold increase in PMN compared to vehicle control), which was significantly lower than that reported for other ‘inert’ nanoparticle types tested in similar models. For example, PMN numbers following administration of 50 nm unmodified polystyrene nanoparticles (50 μg/lung) and hydrophobic polyvinylacetate-co-alcohol nanoparticles (150 nm diameter, 20 μg/lung) were ~ 75-fold higher than the elevated PMN numbers observed for the high dose albumin nanoparticles in this study [44]. Histopathology evaluation identified minor epithelial damage following administration of the highest dose of particles, and some mild cell infiltration was observed following administration of 20 μg/mouse which was not observed in BAL analysis. Further biocompatibility studies using doses between 20 and 400 μg/mouse (~ 1–16 mg/kg) and particularly multiple-dose biocompatibility studies would be the next step towards establishing a no observed adverse effect level (NOAEL) for albumin nanoparticles.

SPECT/CT imaging and post-mortem organ biodistribution studies both showed that albumin nanoparticles remained in the lungs longer than when delivered as a solution. The difference in particle size between albumin in solution (~ 7 nm) and the nanoparticulate form (~ 190 nm) provides an obvious basis for this difference in clearance rates. In its native state, albumin can exploit endogenous mechanisms for protein clearance from the lungs [45], which may not be accessible to nanoparticulate albumin, due to conformation or size. Bondesson et al. [46] determined the clearance rate of ^99m^Tc-labelled macro-aggregated albumin (Nanocoll®), particle size 8–80 nm, from the human lungs (t_1/2_ = 8.75 h) to be considerably slower than that which had been recorded by other groups for albumin delivered in solution (~ 35% cleared in 1 h) [47]. The difference in formulation was determined to be a likely factor for the different clearance rates observed, and may also be a reasonable explanation for the results of this study. The absence of major nanoparticle accumulation in secondary organs allayed concern about potential secondary toxicity which has been raised for other nanoparticle formulations [41], [48], [49]. There was a small but significant increase in albumin-associated activity in the liver and kidneys at 24 and 48 h post-dosing with solution compared to treatment with albumin nanoparticles. This was attributed to smaller nanoscale species (< 10 nm) reaching secondary organs after administration to the lungs through passage into the bloodstream [10]. Mucociliary clearance could not be characterized reliably within the first 24 h of the study, because the fraction of the lung dose cleared *via* this mechanism could not be distinguished from the ~ 60% of the initial dose that was swallowed during o.a. administration. SPECT/CT images showed very little GI tract signal activity at 24 and 48 h post-administration, however, terminal end-point organ biodistribution studies revealed low levels of activity (4–6% of that remaining in the body) in the intestines at these time points, possibly indicating further clearance of a fraction of the remaining lung dose *via* the mucociliary route.

Clearance rate from the lungs is a major influence on both the pharmacokinetic profile of the drug administered in an inhaled nanomedicine, as well as the potential for lung accumulation of the nanoparticle vehicle and may depend on the physicochemical properties of the inhaled nanomaterial. For example, Videira et al. [50] reported that ~ 75% of the lung dose of solid lipid nanoparticles (200 nm) were rapidly cleared from the lungs into lymph in rats within the first hour post-administration. In contrast, Liu et al. [51] reported that polyacrylamide nanoparticles (20–40 nm) were shown to remain primarily in the lungs over 24 h. A recent study by Choi et al. [26] observed that dye-labelled albumin nanoparticles (~ 200 nm) remained in the lungs for much longer periods (> 48 h). As a result of the extended residence time of soluble and nanoparticulate albumin in respiratory tract, studies investigating pulmonary albumin biodegradation must be conducted in order to ensure that multiple dosing will not lead to detrimental accumulation. Unfortunately, the experimental design used in the current study cannot provide detailed insight into this question, as it is not possible to distinguish whether the ^111^In-albumin activity is associated with intact nanoparticles, intact protein or metabolized peptide fragments. Therefore, future studies investigating albumin nanoparticle metabolism, as well as drug release, under conditions that mimic the respiratory tract would be of particular relevance to the development of these systems.

It was interesting to compare the spatial distributions of albumin nanoparticles *versus* solution within the lungs over 48 h, as this is relevant for therapeutic performance of inhaled nanomedicines. Substantial levels of soluble albumin and albumin nanoparticles could be detected within the BAL fluid phase at all time points tested, although the nanoparticle fraction decreased to < 2% of the lung dose by 48 h compared to ~ 7% soluble albumin remaining in the fluid phase at 48 h. This observation provides important information for controlled release strategies, whereby prolonged drug release within compartments of the lungs is intended. Both albumin nanoparticles (~ 6%) and a smaller fraction of soluble albumin (~ 2%) were found within the BAL cellular fraction after 24 h, demonstrating that alveolar macrophages and possibly neutrophils phagocytosize albumin nanoparticles of this size and surface chemistry, suggesting an additional clearance mechanism for these nanoparticles apart from degradation in the lung lining fluid. Finally, the majority of radioactivity in the lung was located in lung tissue with a notable increase in this compartment over time. Migration of soluble and nanoparticulate albumin into the tissue may result from direct uptake into epithelial cells and/or phagocytosis by alveolar macrophages, which then migrate into the lung interstitium [33], [52]. The high tissue retention associated with albumin nanoparticles may be useful for therapeutic regimes such as lung cancer treatment with inhaled nanomedicines [26], which would benefit patients by providing high, sustained drug concentrations in the lung tissue and low systemic drug exposure.

## 5 Conclusions

Administration of albumin nanoparticles to the lungs of mice showed a high biocompatibility over a wide dose range, with evidence of mild inflammation only at the highest dose tested. This data provide a useful basis for the design of multiple dose studies with the aim of establishing a NOAEL value for albumin vehicles. Complimentary longitudinal SPECT/CT imaging and terminal fixed end-point organ biodistribution studies showed that albumin nanoparticles were cleared more slowly and were retained in the lung tissue to a greater extent than the equivalent dose of albumin solution. The biocompatibility, clearance, and biodistribution profiles of both albumin vehicles studied provide useful information for formulation design by allowing researchers to model whether the use of albumin vehicles can achieve a desired pharmacokinetic profile, a suitable administration frequency and sufficient drug loading for realistic development as an inhaled nanomedicine.

## Figures and Tables

**Table 1:** Physicochemical characterization of albumin nanoparticles and serum stability of ^111^In-labelled albumin and albumin nanoparticles over 48 h at 37 °C. Data represent mean ± SD (n = 3–4).

Nanoparticle properties			
	Diameter (nm)	P.D.I.	Zeta potential (mV)
Albumin nanoparticles	108 ± 3	0.125 ± 0.013	− 17.5 ± 1.8
^111^In albumin nanoparticles	193 ± 110	0.279 ± 0.082	− 14.4 ± 0.9


^111^In label retention in FBS at 37 °C (%)		
	24 h	48 h
^111^In albumin solution	97.8 ± 2.1	99.4 ± 0.3
^111^In albumin nanoparticles	98.8 ± 0.6	97.9 ± 0.6
