# Supplementary material for: In vivo biocompatibility, clearance, and biodistribution of albumin vehicles for pulmonary drug delivery
Source: J Control Release. 2015 Jul 28;210:1–9. doi: 10.1016/j.jconrel.2015.05.269 (PMC4674532; doi:10.1016/j.jconrel.2015.05.269)
Supplement: Supplementary file 1 — Supplementary figures. [file mmc1.docx]

**Supplementary Information**

***In vivo* biocompatibility, clearance and biodistribution of albumin vehicles for pulmonary drug delivery**

Woods,^a^ A. Patel,^a,c^ D.Spina,^c^ Y. Riffo-Vasquez,^c^ A. Morgan, ^a,c^ R. T. M. de Rosales, ^b^ K. Sunassee, ^b^ S. Clark, ^b^ H. Collins,^d^ K. Bruce, ^a^ L.A. Dailey^a,e^ and B. Forbes ^a^


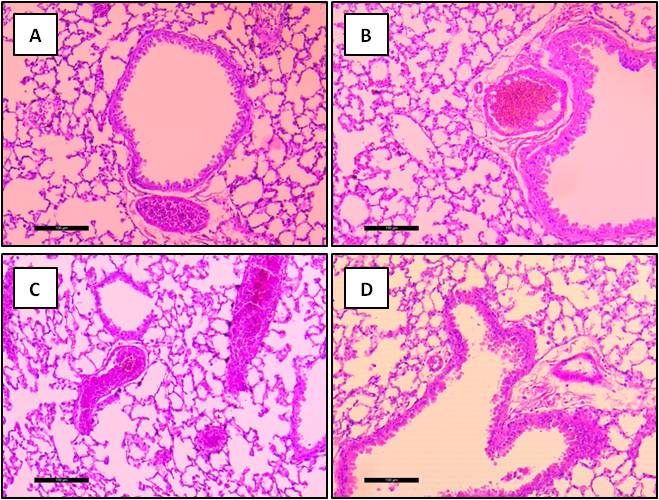


**Figure S1.** Representative histopathological images of a control lung (PBS treated) (A) compared to lung tissue harvested at t=24 h following oropharyngeal administration of 2 µg (B), 20 µg (C) and 390 µg (D) of albumin nanoparticles to the mouse lung (20x magnification; scale bar = 100 µm)


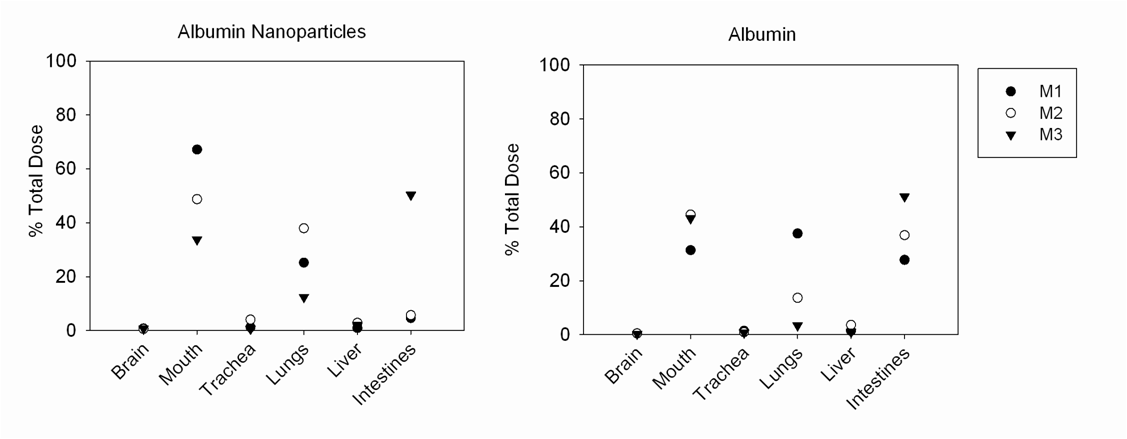


**Figure S2**. Signal distribution of ^111^In-albumin nanoparticles and ^111^In-albumin solution at the first imaging time point following o.a. administration (M1-3 denotes mouse #1-3). Data are decay-corrected values derived from SPECT/CT images and expressed as % total dose.


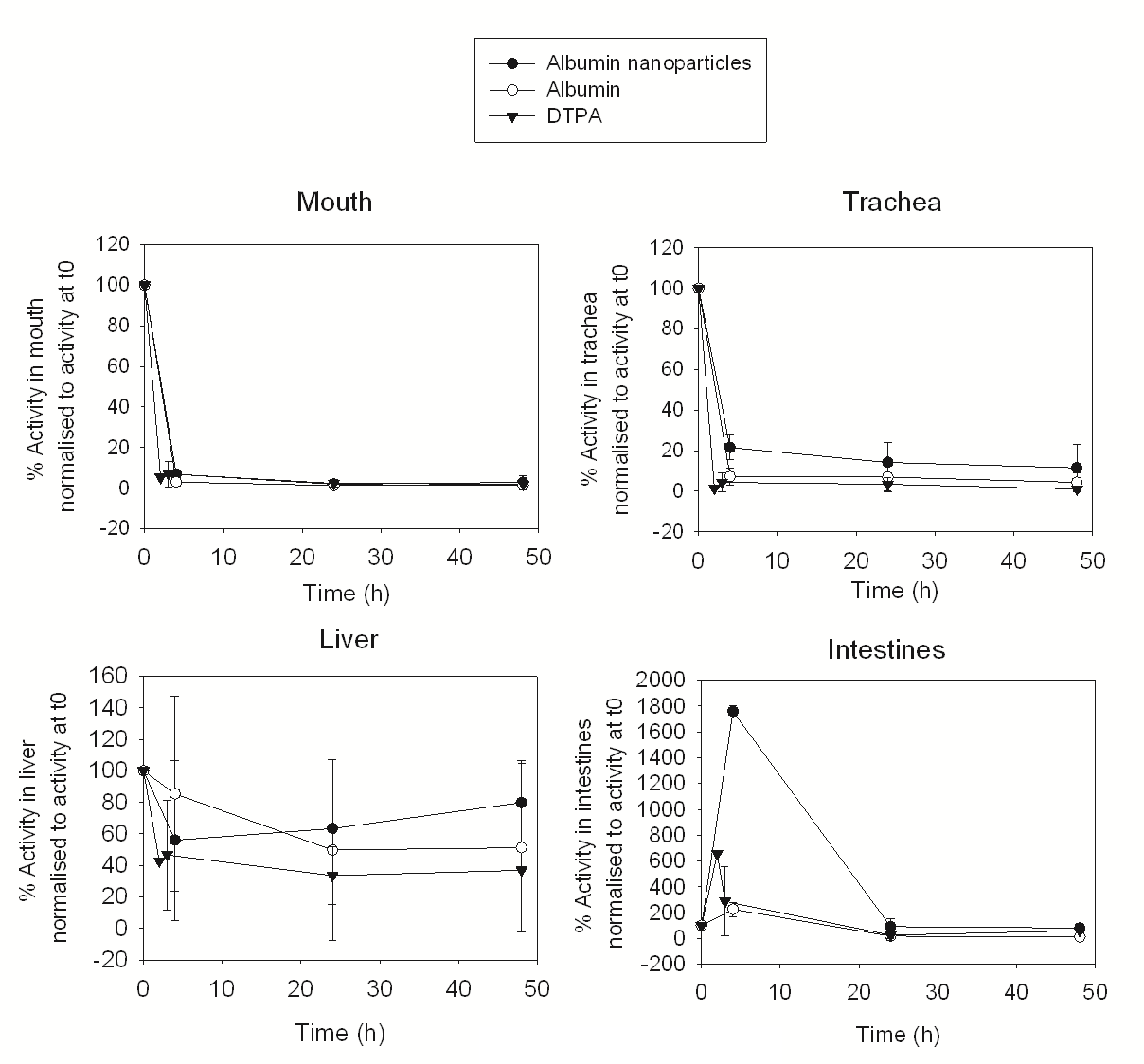


**Figure S3**. Selected organ clearance profiles of ^111^In-albumin nanoparticles (●),^111^In-albumin solution (⭘) and ^111^In-DTPA (▼) following o.a. administration (n=3; mean ± SD). Data are decay-corrected profiles derived from SPECT/CT images and expressed as % ^111^In activity relative to activity at t=0.


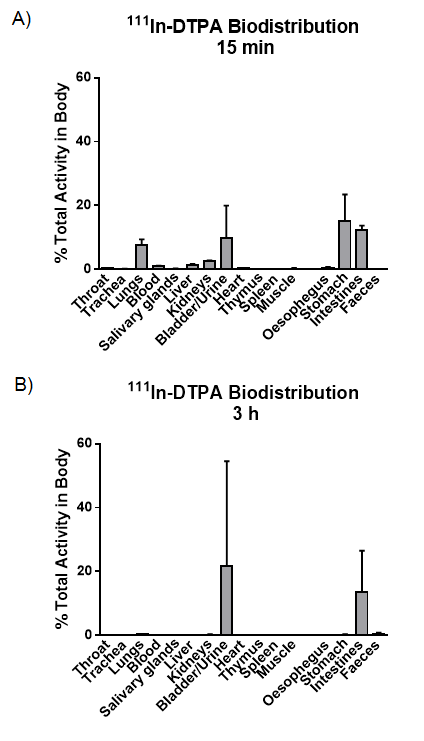
**Figure S4**. ^111^In activity measured directly in major organs of mice expressed as the proportion of total activity remaining in the body at time points t= 15 min and 3 h (n=3). All activity was eliminated from the body by the t = 24 h time point.
